# PLGA sustained-release microspheres loaded with an insoluble small-molecule drug: microfluidic-based preparation, optimization, characterization, and evaluation *in vitro* and *in vivo*

**DOI:** 10.1080/10717544.2022.2072413

**Published:** 2022-05-09

**Authors:** Yue Su, Jia Liu, Songwen Tan, Wenfang Liu, Rongrong Wang, Chuanpin Chen

**Affiliations:** a Xiangya School of Pharmaceutical Sciences, Central South University, Changsha, China; b Hunan Institute for Drug Control, Changsha, China

**Keywords:** Microsphere, microfluidics, insoluble small-molecule drug, poly(lactic-co-glycolic acid), pharmacokinetics

## Abstract

Microspheres play an important role in controlling drug delivery and release rate accurately. To realize the sustainable release of insoluble small-molecule drugs, a new three-phase flow-focusing microfluidic device was developed to produce the drug-loaded sustained-release microspheres which were prepared with bicalutamide (BCS class-II) as the model drug and poly(lactide-co-glycolide) (PLGA) as the carrier material. Under optimized prescription conditions, the microspheres showed a smooth surface and uniform size of 51.33 μm with a CV value of 4.43%. Sustained-release microspheres had a releasing duration of around 40 days *in vitro* without any initial burst release. The drug release mechanism of the microspheres was drug diffusion and polymer erosion. Meanwhile, the drug release of microspheres *in vivo* could be up to 30 days. Briefly, the microfluidic device in this study provides a new solution for the preparation of sustained-release microspheres for insoluble small-molecule drugs. PLGA sustained-release microspheres developed by the microfluidic device have good application prospects in precise delivery and sustainable release of insoluble small-molecule drugs.

## 1. Introduction

At present, most insoluble small-molecule drugs cannot achieve the ideal therapeutic effect due to the limitation of the ADME process *in vivo* from their physical and chemical properties (Kim et al., 2011; Jiang et al., 2014). To achieve a stable effective blood concentration, patients need to take drugs frequently, which is prone to having side effects on physical and mental health because of poor compliance and wide fluctuating blood concentration level (Gaspar et al., 2015; Zhai et al., 2015; Park et al., 2019). Generally, drugs that have very poor water solubility have low bioavailability after administration of common dosage forms, which limits the clinical application (Ma, 2014; Wei et al., 2018; Shah et al., 2019; Lu et al., 2020; Butreddy et al., 2021; Yan et al., 2021). Therefore, it is one of the solutions to find a suitable dosage form for insoluble small-molecule drugs delivery to solve long-term drug delivery problems and improve bioavailability.

The microsphere is a promising option, which is a kind of microparticle dispersion system formed by adsorbing or dispersing drugs in the polymer material. As an advanced delivery system, microspheres have been widely used in many fields, such as food, drug delivery, and cosmetics (Kim et al., 2012; Diarrassouba et al., 2015; Da Costa Neto et al., 2019; Hao et al., 2019). The function and application of microspheres have been actively explored. In the biomedical field, it is generally believed that the microsphere as a delivery carrier can store and deliver drugs with different properties and molecular weights. Meanwhile, the diversity of microsphere structure and material selection make them have advantages in controlling drug release rate and location (Ni et al., 2020; He et al., 2021; Su et al., 2021). Most importantly, the microspheres can significantly prolong the duration of drug treatment and reduce side effects. The preparation of microsphere loaded with insoluble small-molecule drugs has always been a research trend. However, only two of the 12 microsphere products approved by the U.S. Food and Drug Administration (FDA) are suitable for insoluble small-molecule drugs at present (Lee et al., 1997; Allen & Evans, 2020), which indicates the difficulties of developing microspheres loaded with insoluble small-molecule drugs including uneven particle size, poor sustained release effect, and complex operation. Therefore, it is necessary to explore the preparation and prescription of microspheres loaded with insoluble small-molecule drugs and to study their *in vitro* drug release effect and *in vivo* pharmacokinetic characteristics to ensure the effectiveness of their practical application.

Microspheres have strict requirements for particle size and uniformity. The uniformity of microsphere size has an important influence on the controllability of drug release position and release rate (Iqbal et al., 2015; Ramazani et al., 2015). What is more, it is necessary to adjust the size of microspheres for adapting to the standards of drug administration for different diseases. Traditional preparation methods of microspheres usually have wide particle size distribution, such as emulsification and spray drying (Arrighi et al., 2019; Steipel et al., 2019; Hsu et al., 2020). Therefore, it is very important to develop a technology that can accurately control the particle size and size distribution of microspheres. In recent years, microfluidic technology as new technology has been used in microspheres preparation. This novel technology can control the flow and mutual shear of microfluidics on a microfluidic device to produce microdroplets with uniform and controllable particle size and monodispersity (Li et al., 2018; Rezvantalab & Keshavarz Moraveji, 2019). Microfluidic technology has a great development space in the field of microspheres. In addition to achieving uniform and controllable particle size, the internal structure of the microspheres can also be customized to develop microspheres with ideal pharmacokinetic curves and loading capacity (Peng et al., 2020).

There are many kinds of materials for preparing microspheres, and poly(lactide-co-glycolide) (PLGA) is one of the most popular carrier materials (Jiang et al., 2005; Lu et al., 2009). It is a copolymer composed of lactic acid (LA) and glycolic acid (GA), which is mainly degraded by hydrolysis *in vivo* and discharged in the form of water and carbon dioxide after the tricarboxylic acid cycle. Therefore, PLGA has good biocompatibility and biodegradability (Choi et al., 2021; Wen et al., 2022), and its safety has been recognized by FDA and the European Drug Agency, and has been officially included by FDA as pharmaceutical excipients. Moreover, PLGA also has the characteristics of easy processing and suitable biodegradation kinetics (Kim et al., 2021). The hydrophilic and hydrophobic properties and degradation kinetics of PLGA can be changed by adjusting the monomer composition ratio (LA/GA), molecular weight, and terminal groups of PLGA (Hua et al., 2021). Therefore, PLGA is a promising carrier material for microspheres preparation.

In this paper, we used microfluidic technology to prepare PLGA sustained-release microspheres loaded with small-molecule drugs. Bicalutamide (BCL) for the treatment of prostate cancer was selected as the model drug. BCL belongs to the BCS class-II and is mainly administered orally as tablets (Ray et al., 2017). The absorption into circulation through the intestinal epithelium is limited due to poor water solubility (0.005 mg/mL). First, a three-phase focused microfluidic device was designed to prepare BCL-loaded PLGA microspheres, and the formulation of BCL-loaded PLGA microspheres was optimized. Next, the optimized BCL-loaded PLGA microsphere was evaluated through a series of characterization, including particle size analysis, morphology, drug loading (DL%), entrapment efficiency (EE%), Fourier-transform infrared spectroscopy (FTIR), and differential scanning calorimetry (DSC). Finally, *in vitro* release behavior and *in vivo* pharmacokinetics of BCL-loaded PLGA microspheres were studied.

## 2. Materials and methods

### 2.1. Materials

Poly(lactide-co-glycolide) (Resomer^®^ RG 504, lactide:glycolide 50:50, ester terminated Mw 38,000–54,000) was purchased from Sigma-Aldrich (St. Louis, MO). Poly(vinyl alcohol) (PVA) was purchased from Tianjin Kemel Chemical Reagent Co., Ltd. (Tianjin, China). Bicalutamide was obtained as a gift sample from Zhendong Pharmaceutical Co., Ltd. (Shanxi, China). Sylgard 184 silicone elastomer kits including polydimethylsiloxane (PDMS) and the curing agent were purchased from Dow Corning Co., Ltd. (Midland, MI). Dichloromethane (DCM) and tetrahydrofuran (THF) were purchased from Sinopharm Chemical Reagent Co., Ltd. (Shanghai, China). All other chemicals and reagents were of analytic grade and purchased from Sinopharm Chemical Reagent Co., Ltd. (Shanghai, China).

### 2.2. Design and preparation of the microfluidic device

**Figure 1.**
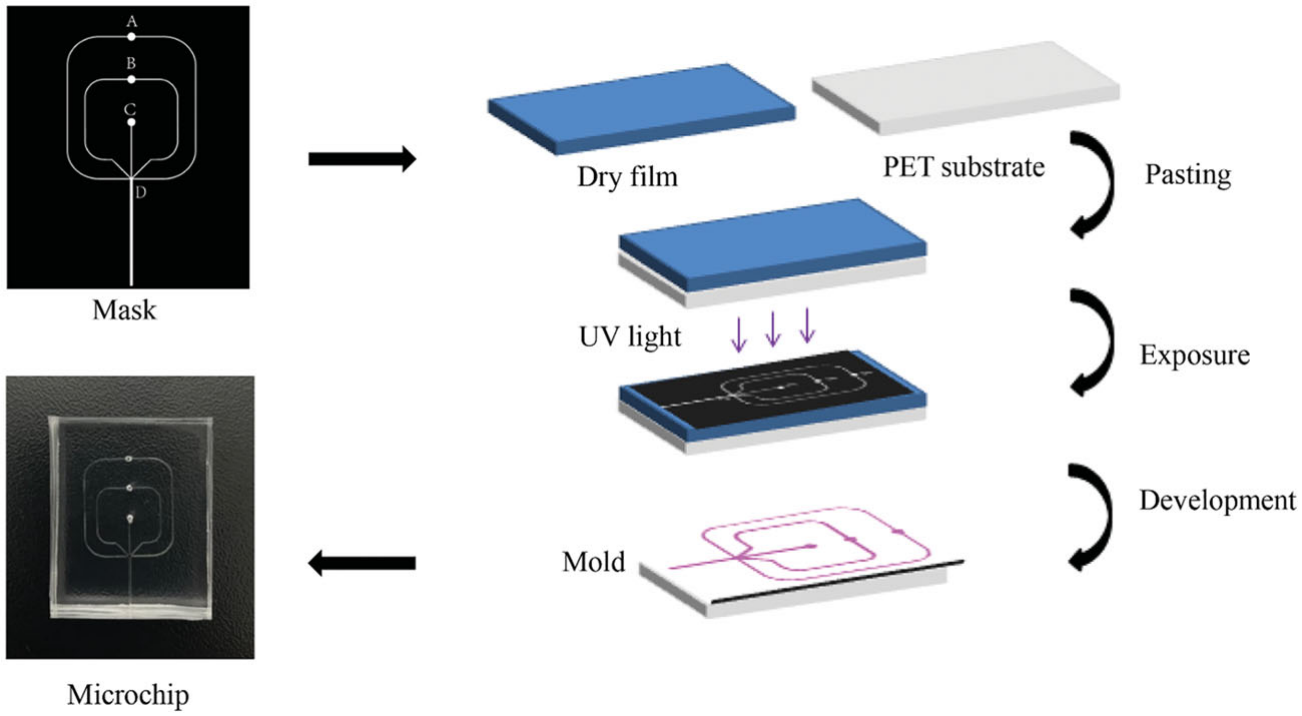
Schematic diagram of the microfluidic device preparation process.

The microfluidic device was manufactured by standard lithography and soft lithography (Zeng et al., 2020). A schematic diagram of the process is shown in Figure 1. First, Adobe Illustrator was used to complete the design of the microchannel structure. As shown in the mask (Figure 1), the depth and width of the microchannel at inlets A, B, and C were 100 μm, and the width and depth of the microchannel at outlet D were 350 μm and 100 μm, respectively. Then, dry film (Riston FX900, DuPont Co., Ltd., Wilmington, DE) was used as raw material to prepare microchannel molds. Dry film was pasted on poly-ethylene terephthalate (PET) film (0.175 mm, Kaivo Optoelectronic Technology Co., Ltd., Zhuhai, China) and exposed to ultraviolet (UV) (HT-3040, Shijiazhuang Ourpcb Co., Ltd., Shijiazhuang, China) for 120 s. Next, the exposed dry film was immersed in 1 wt% Na_2_CO_3_ solution for development. Finally, PDMS prepolymer and curing agent were mixed in the ratio of 10:1 and then cast on the mold for curing for 2 h (60 °C). After plasma treatment, the cured PDMS chip containing microchannels was tightly bonded with a blank PDMS chip.

### 2.3. Preparation of drug-loaded PLGA microspheres

**Figure 2.**
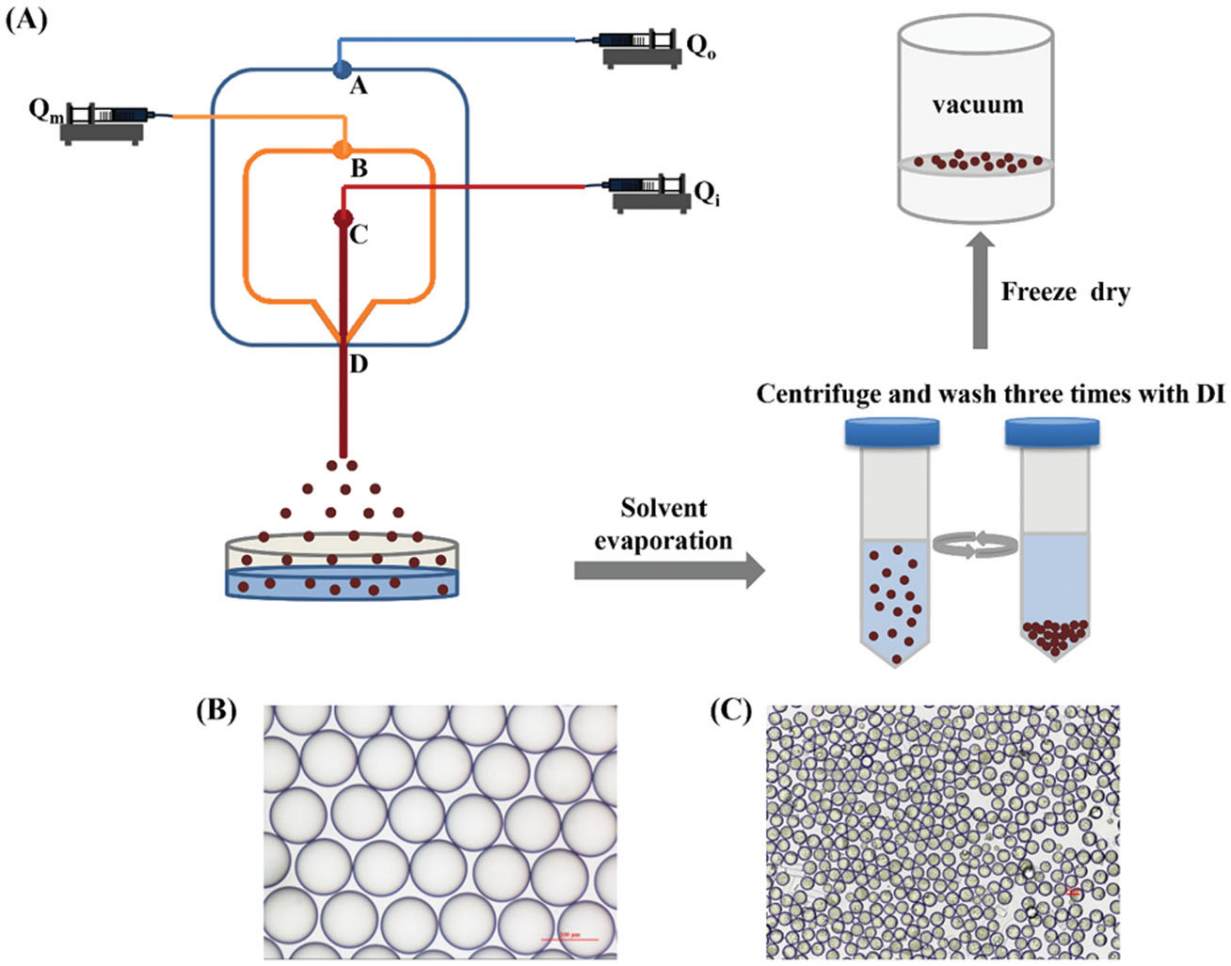
(A) Schematic diagram of drug-loaded PLGA microspheres preparation process. (B) Optical photograph of microdroplets prepared via the microfluidic device. (C) Optical photograph of drug-loaded PLGA microspheres prepared via the microfluidic device.

Drug-loaded PLGA microspheres were prepared by a three-phase flow-focusing microfluidic device. PLGA solution containing BCL (dissolved in the mixture of DCM and acetone) was used as the innermost oil phase (*Q*_i_). The middle oil phase (*Q*_m_) was PLGA solution (dissolved in DCM). PVA aqueous solution (1 wt%) was used as outer continuous phases (*Q*_o_). As shown in Figure 2(A), the injection pump (LSP01-1A, Longer Precision Pump Co., Ltd., Baoding, China) was used to import *Q*_o_, *Q*_m_, and *Q*_i_ into microchannels of the microfluidic chip from the inlets A, B, and C, respectively. By adjusting the flow rate ratio of each phase, O/O/W microdroplets formed at the intersection of microchannels due to shear force. The flow rates of *Q*_o_, *Q*_m_, and *Q*_i_ controlled by the syringe pump were 500, 65, and 50 μL/min, respectively. PVA aqueous solution (1 wt%) in a container modified with PVA was placed at outlet D for collecting microdroplets. The collected microdroplets were placed in an incubator and solidified to form PLGA microspheres by evaporation of DCM and acetone (37 °C, 24 h). The solidified microspheres were collected by centrifugation (5000 rpm, 5 min, Beckman Coulter, Brea, CA) and then washed with deionized (DI) water three times to remove residual PVA on the surface of the microspheres. All these microspheres were freeze-dried (LC-10N-80A, Lichen Co., Ltd., Shanghai, China) for 24 h.

### 2.4. Characterization of BCL-loaded PLGA microspheres

#### 2.4.1. Particle size analysis

The mean size and size distribution were evaluated by Image J (Bethesda, MD) (He et al., 2021). The coefficient of variation (CV) was calculated from statistical data based on the following equation:

$$\mathrm{CV}=\frac{r_{\mathrm{sd}}}{D_{m}}\;(1)$$

where *r*_sd_ is the standard deviation of diameter (μm) and *D*_m_ is the mean diameter (μm).

#### 2.4.2. Appearance and morphological characterization of BCL-loaded PLGA microspheres

The size, shape, and morphology of the freeze-dried microspheres were observed by scanning electron microscope (JEOL, JSM-7800F, Tokyo, Japan). The sample was attached to the sample holder with double-sided adhesive and sputtered with gold before observation.

#### 2.4.3. Differential scanning calorimetry

The thermal properties of BCL, PLGA, their physical mixtures, and BCL-loaded PLGA microspheres were determined by a differential scanning calorimeter (HSC-4, Beijing, China). Accurately weighed samples were individually sealed in a standard aluminum pot. The scanning temperature range of DSC was 30 °C to 235 °C and the heating rate is 10 °C/min.

#### 2.4.4. Fourier-transform infrared spectroscopy

FTIR spectra were analyzed using an FTIR spectrophotometer (Shimadzu 8300, Tokyo, Japan). Samples of BCL powder, PLGA, and BCL-loaded PLGA microspheres were scanned from 400 to 4000 cm^−1^. The pellet was prepared using the KBr method.

#### 2.4.5. Determination of entrapment efficiency and drug loading

The EE% and DL% of BCL-loaded PLGA microspheres were analyzed by high-performance liquid chromatography (HPLC, Acquity Arc, Waters Co., Ltd., Milford, MA). Briefly, a known mass of BCL-loaded PLGA microspheres was dissolved in THF. The sample was filtered through a 0.22 μm filter (Jinteng Laboratory Equipment Co., Ltd., Tianjin, China) after ultrasonic breakdown and dissolution and then injected into an HPLC system to determine the concentration of BCL. The determination was performed on a Kromasil C18 column (250 mm × 4.60 mm, Nouryon, Amsterdam, Netherlands) with the column temperature maintained at 30 °C. The mobile phase was 0.1% potassium dihydrogen phosphate aqueous solution/acetonitrile (47:53, v/v) and was delivered at a flow rate of 1 mL/min. The detection wavelength was 270 nm and the injection volume was 10 μL. All measurements were conducted in triplicate and the results were expressed as mean ± SD. Finally, the DL and EE were calculated by the following equations:

$$\mathrm{DL}\;(\%)=\frac{w_{1}}{w_{2}}\times 100\%\;(2)$$

$$\mathrm{EE}\;(\%)=\frac{\text{actual DL}}{\text{theoretical DL}}\times 100\%\;(3)$$

where *W*_1_ and *W*_2_ represent the weight of the drug in microspheres and the weight of the whole microsphere, respectively. Results are expressed as mean ± SD (*n* = 3).

### 2.5. *In vitro* release profile and degradation of BCL-loaded PLGA microspheres

During the release test, a mass of BCL-loaded PLGA microspheres was placed in a pretreatment dialysis bag (MW = 3500 Da). Then, the dialysis bag was immersed in 30 mL of isotonic phosphate buffer saline (PBS) solution (pH 7.40) containing SDS. Temperature and speed were maintained by the incubator (37 °C, 100 rpm, SPX-100B-D, Boxun Industrial Co., Ltd., Shanghai, China). 1.50 mL medium was extracted at regular intervals and the same amount of fresh medium was added. The extracted samples were filtered with a 0.22 μm filter. The samples were injected into HPLC for quantitative analysis. All measurements are performed in triplicate and the results are expressed as mean ± SD (*n* = 3).

To explore the mechanism of drug release from PLGA microspheres, the collected *in vitro* release data were fitted to the following release kinetic models, including zero-order (4), first-order (5), Higuchi (6), and Korsmeyer-Peppas (7) models.

$$Q=A+K^{*}\;T\;(4)$$

$$Q=A^{*}(1-\exp\left(-K^{*}T\right))\;(5)$$

$$Q=A+K^{*}T^{1/2}\;(6)$$

$$Q=A+K^{*}T^{n}\;(7)$$

where *Q* represents the cumulative release of drug in time, *A* is the constant, *K* is the kinetic constant, and *n* is the diffusion constant.

During the *in vitro* degradation experiment, the sample was pretreated in the same way as in the release experiment. PLGA microspheres were taken out every certain time, then centrifuged, washed with distilled water, freeze-dried, and SEM was used to observe the changes of PLGA microspheres with time.

### 2.6. *In vivo* pharmacokinetic study

#### 2.6.1. Animals and drug administration

Male healthy New Zealand rabbits weighing approximately 2.0 kg were provided from Taiping Biotechnology Co., Ltd. (Hunan, China). All rabbits were subjected to a 12 h light–dark cycle in a room controlled by temperature and relative humidity. During adaptation, rabbits were allowed free access to water and standardized food. After three days of adaptation, rabbits were fasted for 12 h before administration but were allowed to drink freely during the period. The study conformed to the Guide for the Care and Use of Laboratory Animals.

Twelve rabbits were randomly divided into two groups with six rabbits in each group. Group I was an oral group, which was administered with BCL aqueous suspension (2.73 mg/kg) by gavage. Group II received a single intramuscular injection of BCL-loaded PLGA microspheres. The microspheres were resuspended in microsphere diluent (0.66% NaCl, 0.63% CMCNa, 0.02% Tween 80) before injection. The microsphere suspension was injected into the thigh muscle of the rabbit’s hind leg (BCL dose: 2.73 mg/kg). After injection, the blood samples of the ear vein were collected in heparinized test tubes at regular intervals in both groups. All blood samples were immediately centrifuged at 3000 rpm for 10 min, then plasma samples were obtained and stored at −80 °C for analysis.

#### 2.6.2. Plasma sample analysis

The concentration of BCL in plasma was determined by HPLC. Eight microliters plasma sample was taken and 2.4 mL methanol was added, then vortex mixed for 3 min, and centrifuged at 9500 rpm for 15 min before separating the supernatant. The supernatant was dried with nitrogen at 60 °C. The dry residue was then reconstituted with 150 μL methanol. The samples were filtered with a 0.22 μm organic membrane and injected into the HPLC system for analysis.

#### 2.6.3. Pharmacokinetic data analysis

WinNonlin 6.1 software was selected to process plasma concentration data at different time points. Relevant pharmacokinetic parameters were analyzed with the non-compartmental model, including the maximum plasma drug concentration (*C*_max_) and the time required to reach *C*_max_ (*T*_max_), the area under the plasma concentration–time curve (AUC), mean residence time (MRT), apparent volume of distribution (Vz), and plasma clearance (Cl).

## 3. Results and discussion

### 3.1. Preparation of BCL-loaded PLGA microsphere

BCL-loaded PLGA microspheres were prepared by a three-phase focused microfluidic device. The effects of the volume ratio of innermost phase (*Q*_i_) solvent DCM and acetone, the concentration of BCL, and the concentration of PLGA in different phases on DL% and EE% were studied.

**Figure 3.**
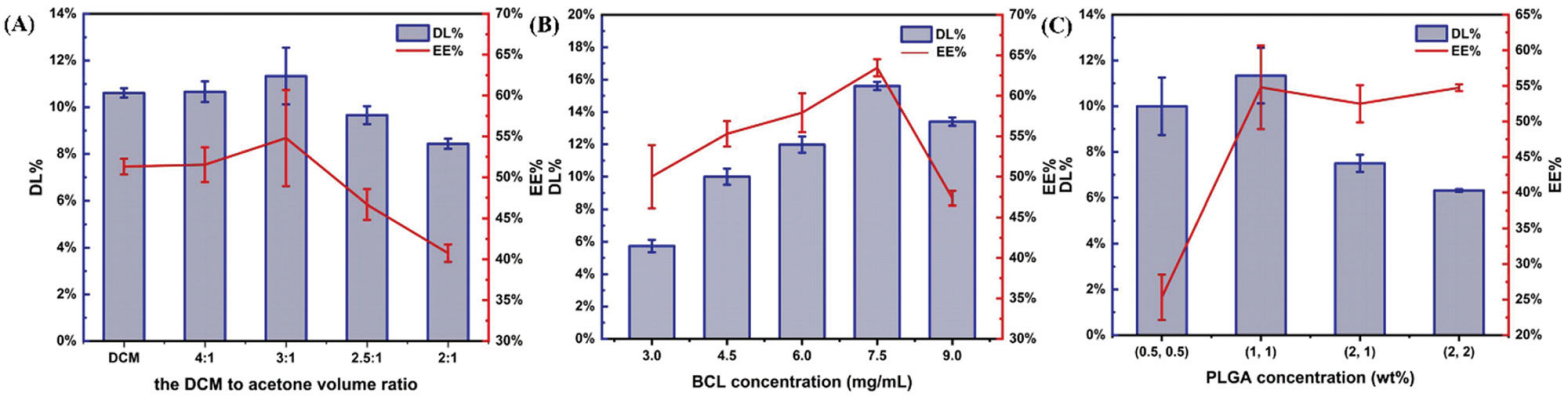
Effects of parameters on drug loading and entrapment efficiency of microspheres (*n* = 3). (A) The effects of dichloromethane to acetone (D/A) volume ratio. BCL concentration was 7.5 mg/mL and PLGA concentration of different phases was 1 wt%. (B) The effects of BCL concentration. The D/A volume ratio was 3:1 and the PLGA concentration was 1 wt%. (C) The effects of PLGA concentration. The D/A volume ratio was 3:1 and the BCL concentration was 7.5 mg/mL.

The effects of dichloromethane to acetone (D/A) volume ratio on EE% and DL% were investigated when BCL concentration was 7.5 mg/mL and PLGA concentration was 1 wt%. As shown in Figure 3(A), with the decrease of the D/A volume ratio, the EE% and DL% of microspheres first increased and then decreased. When the D/A volume ratio was 3:1, DL% and EE% of microspheres were the highest. BCL was slightly dissolved in DCM, and DL% and EE% of the microspheres prepared were low when only DCM was used as the solvent. When acetone was added, DL% and EE% increased as the ratio of the D/A volume ratio decreased, because acetone was beneficial to the dissolution of BCL. However, acetone is a miscible solvent with water. When the volume of acetone increased to a certain level, part of the drug could diffuse into the water phase with acetone, resulting in the reduction of the drug content in the microspheres.

Next, the influence of BCL concentration on EE% and DL% was further studied under the conditions that the D/A volume ratio was 3:1 and PLGA concentration was 1 wt%. BCL concentration varied from 3.0 to 9.0 mg/mL. As shown in Figure 3(B), the DL% and EE% of microspheres increased first and then decreased with the increase of BCL concentration. When the concentration of BCL was 7.5 mg/mL, DL% and EE% were the highest. The reason might be that the increase of DL% and EE% was not proportional to the increase of initial drug concentration (Song et al., 2008). Therefore, DL% and EE% began to decline when the drug concentration increases to a certain level.

Furthermore, the effects of PLGA concentration on DL% and EE% were investigated when the D/A volume ratio was 3:1 and BCL concentration was 7.5 mg/mL. Figure 3(C) shows the trend of DL% and EE% as PLGA concentration changes. The results showed that DL% and EE% of the microspheres first increased with the increase of PLGA concentration, but when PLGA concentration reached a certain concentration, EE% did not increase and DL% began to decrease. This phenomenon might be caused by the increase of viscosity of the oil phase because PLGA concentration increased. Increasing the viscosity reduced the diffusion of the drug into the aqueous phase (Song et al., 2008; Jiang et al., 2014). However, the particle size of the microspheres would gradually increase with the increase of PLGA concentration (Jeong et al., 2008). The hydrophobic drug molecules were likely to be more easily adsorbed on the surface of the microspheres with large particle sizes, resulting in the reduction of drug content in the microspheres (Song et al., 2008).

In a word, the experimental parameters were optimized as follows: BCL concentration was 7.5 mg/mL, PLGA concentration was 1 wt%, and D/A volume ratio was 3:1. The DL% and EE% of PLGA microspheres prepared under the optimized formula were 15.60 ± 0.26% and 63.47 ± 1.05%.

### 3.2. Characterization of BCL-loaded PLGA microspheres

**Figure 4.**
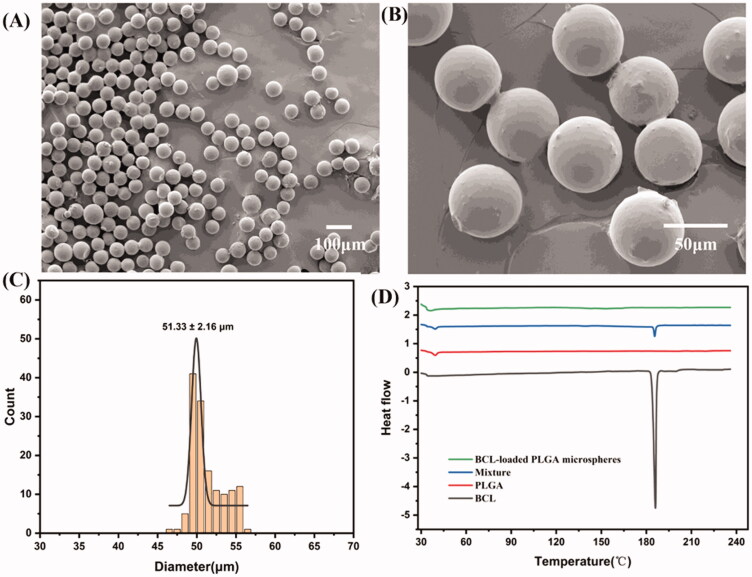
(A, ×100 and B, ×500) SEM images of BCL-loaded PLGA microspheres prepared by a three-phase focused microfluidic device. (C) Particle size distribution. (D) DSC curves of BCL, PLGA, mixture, and BCL-loaded PLGA microspheres.

Morphology of the obtained PLGA microspheres was observed by scanning electron microscope (SEM). As shown in Figure 4(A,B), the microspheres were spherical with smooth surfaces, highly monodisperse, and had no aggregation.

The size distribution of microspheres is shown in Figure 4(C). The results showed that the average diameter of PLGA microspheres was 51.33 ± 2.26 μm under the current fixation conditions, which could achieve a good sustained release effect of the encapsulated drugs *in vivo*. The CV of microspheres was 4.21%, which indicated that the microspheres had a narrow particle size distribution.

Differential scanning calorimetry is commonly used to analyze the physical state of drugs encapsulated in microspheres. As shown in Figure 4(D), BCL showed a typical absorption peak at 186.1 °C. PLGA had an absorption peak at 38.6 °C. The two characteristic peaks of the physical mixture corresponded to the absorption peaks of BCL and PLGA, respectively. Only one absorption peak appeared in BCL-loaded PLGA microspheres at 36.2 °C, but the absorption peak of BCL disappeared (Zhai et al., 2015). The results showed that BCL was encapsulated in microspheres and the drug in microspheres existed in an amorphous form.

**Figure 5.**
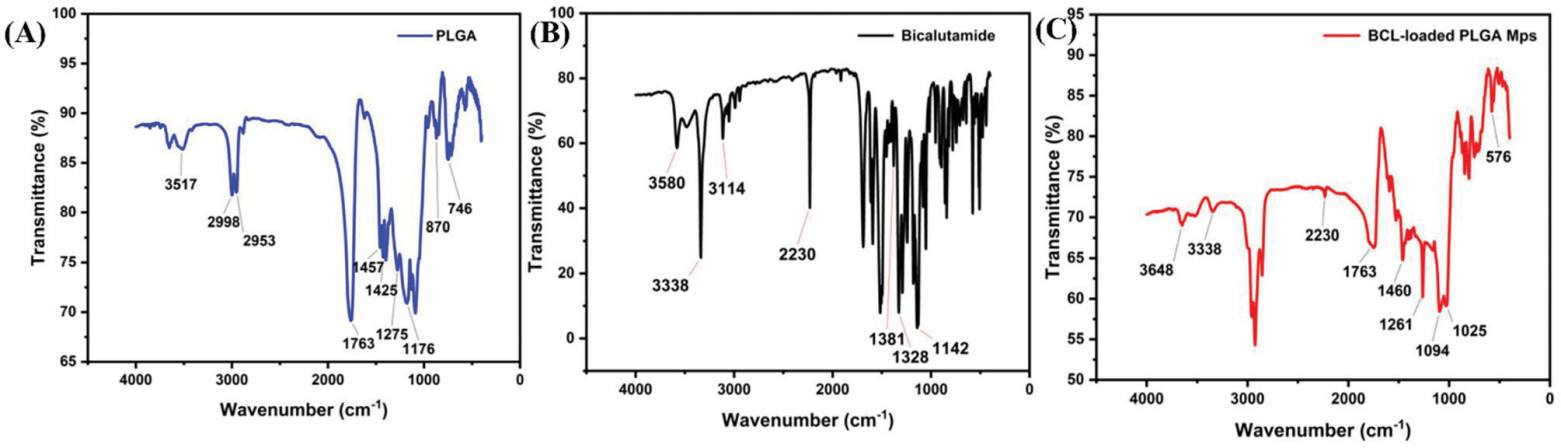
FTIR spectra of (A) PLGA, (B) BCL, and (C) BCL-loaded PLGA microspheres.

To study the interaction between drug and polymer, FTIR was performed on the microspheres prepared under the optimized formulation and the spectra were compared with those of PLGA and BCL. Figure 5 shows FTIR spectra of PLGA (A), BCL (B), and microspheres (C). Characteristic peaks were observed at 3338 cm^−1^, 3114 cm^−1^, and 2230 cm^−1^ in BCL spectrum, and 3517 cm^−1^ and 1763 cm^−1^ in PLGA spectrum. These peaks of PLGA and BCL were also observed in the prepared microspheres, confirming that no additional covalent bonds were formed during the preparation process.

### 3.3. *In vitro* release profile of BCL-loaded PLGA microsphere

**Figure 6.**
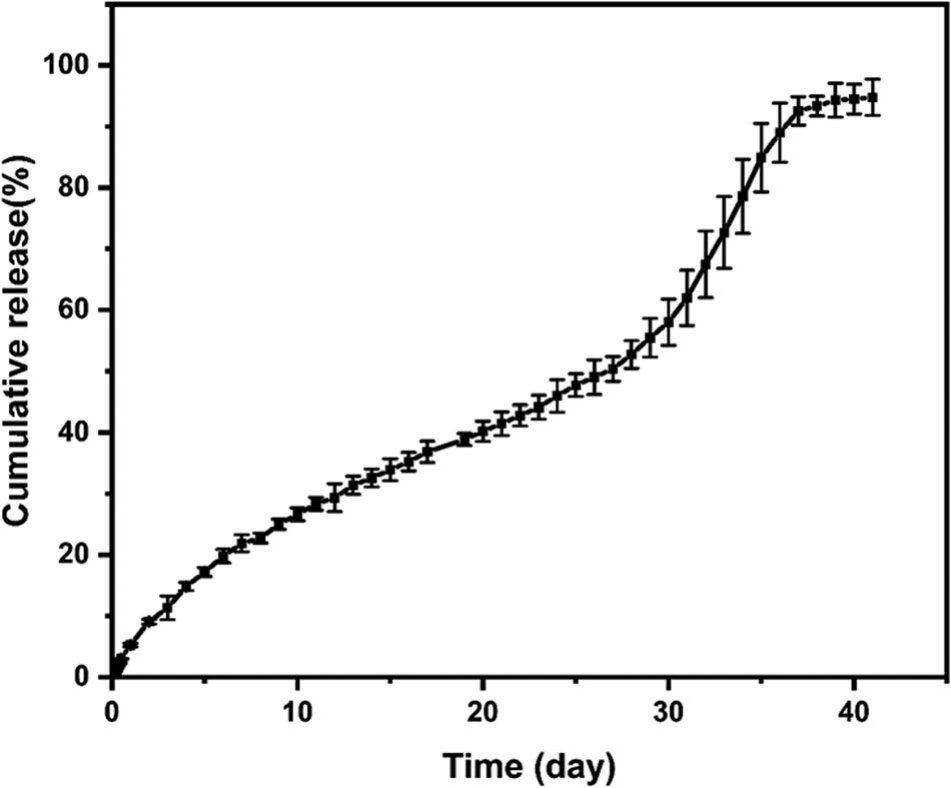
*In vitro* release profile of BCL-loaded PLGA microspheres. Data represent mean ± SD (*n* = 3).

The release of encapsulated drugs in PLGA microspheres *in vitro* was evaluated and the degradation of PLGA microspheres was monitored. Figure 6 shows the *in vitro* release curve of BCL in PLGA microspheres. BCL was continuously released from PLGA microspheres for up to 40 days *in vitro*. The release of drug-loaded PLGA microspheres can be divided into three stages: the first stage was 0–27 days, the release rate was relatively slow, and the cumulative release of BCL was 50.33%. The second stage lasted from 27 to 37 days, the release rate was accelerated, and the cumulative release of BCL reached 92.55%. The third stage was 37–40 days, during which the cumulative release of BCL tended to be stable and the curve changed almost constantly. The maximum release rate of BCL was 94.78% on the 40th day.

To investigate the mechanism of BCL release from PLGA microspheres, *in vitro* release data were fitted to zero-order, first-order, Higuchi, and Korsmeyer-Peppas models. The results of related parameters obtained by linear regression for each kinetic model are shown in Table 1. The results showed that Korsmeyer-Peppas models had the highest correlation coefficient *R*^2^ and thus showed the best fit with *in vitro* release data. Moreover, the *n* value was 0.76 (0.43<*n* < 0.85), indicating a non-Fick diffusion kinetics (Zhou et al., 2021). It could be concluded that the drug release mechanism was mainly affected by drug diffusion and skeleton erosion.

**Table 1.** Fitting results of different dynamic models on BCL-loaded PLGA microsphere.

Model	*R* ^2^
Zero-order model	0.9619
First-order model	0.9605
Higuchi model	0.8990
Korsmeyer-Peppas model	0.9748

In the first stage of *in vitro* release, the drug release rate was slow. Since the degradation of PLGA was not obvious, the drug may be released mainly by diffusion from the inside of the polymer (Chen et al., 2018). The thickness of the PLGA layer has a great influence on the release rate in this stage. According to a previous study (He et al., 2021), the thickness of the PLGA layer could be adjusted by changing the flow velocity of the middle phase in a three-phase focused microfluidic chip. The release rate of the thick PLGA layer was slower than that of the thin PLGA layer. Moreover, there was no initial burst release of BCL-loaded PLGA microspheres in the first 24 hours, and the cumulative release was only 5.25%, mainly due to the small amount of BCL attached to the surface of microspheres. Microfluidic technology and acetone both contributed to a no initial burst release (He et al., 2021). Acetone helped dissolve BCL. Due to the higher ratio of DCM to acetone (3:1, v/v) and the much lower boiling point of acetone than DCM, DCM evaporated faster than acetone at room temperature. Therefore, BCL diffusion to the surface of microspheres with acetone might be limited during microsphere solidification, so BCL was largely encapsulated in microspheres probably (Shen et al., 2015). In the second stage, the surface porosity of microspheres increased with the hydrolysis and erosion of PLGA. Therefore, the release of drugs from the inside of the microsphere was accelerated. Release at this stage was mainly controlled by PLGA degradation and erosion. In the third stage, as the drug concentration inside and outside the dialysis bag tended to balance, the drug release rate became slow again and gradually reached the plateau stage.

**Figure 7.**
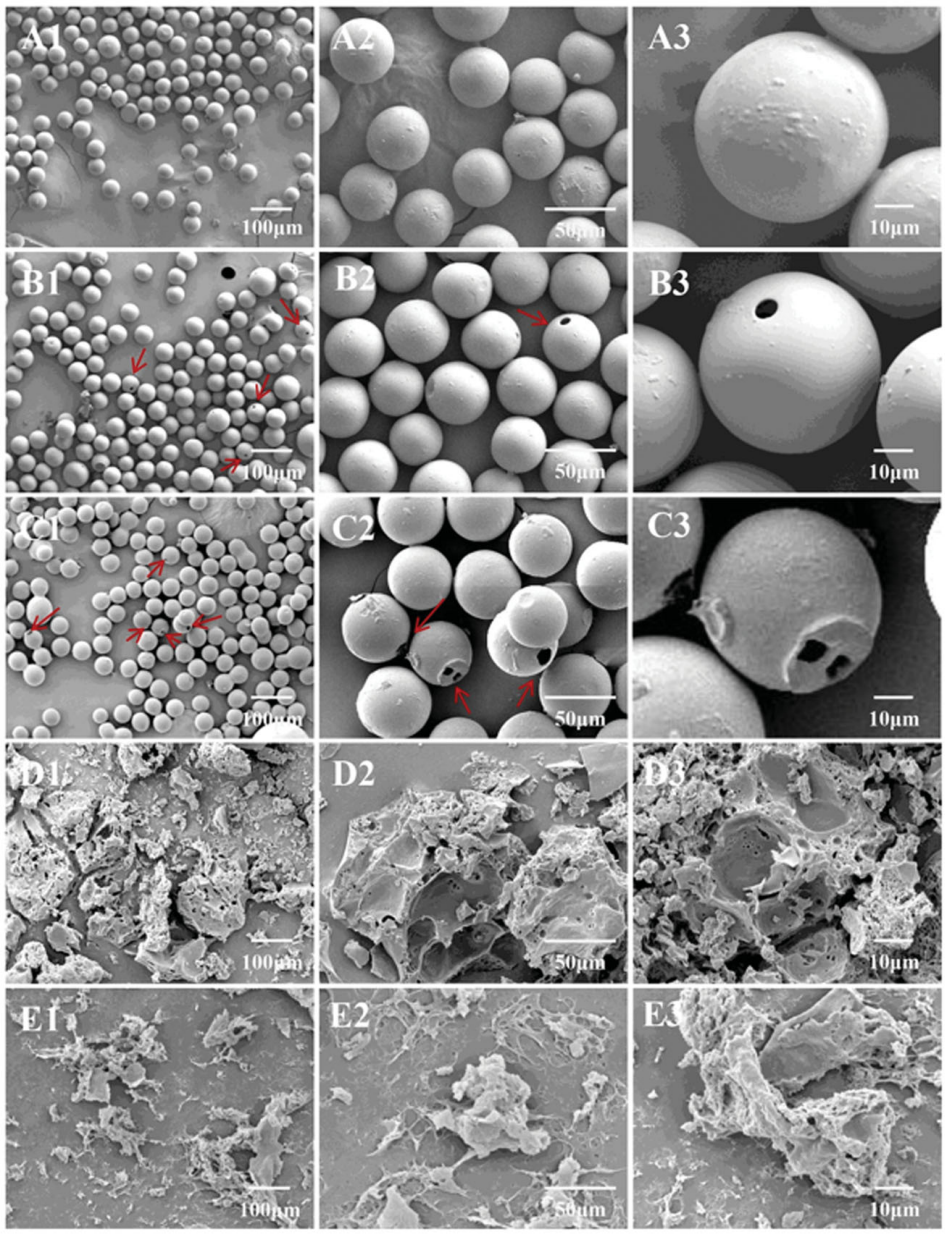
Morphological change of PLGA microspheres during *in vitro* degradation. (A1–A3) 1 week; (B1–B3) 3 weeks; (C1–C3) 5 weeks; (D1–D3) 7 weeks; and (E1–E3) 9 weeks. The arrows point to PLGA microspheres with holes.

The degradation of microspheres with time was observed by SEM. As shown in Figure 7(A1–A3), the morphology of the microspheres was not significantly different from that before preparation after one week of degradation *in vitro*. However, small holes began to appear on the surface of a small number of microspheres after 3 weeks (Figure 7(B1–B3)). With time to the fifth week (Figure 7(C1–C3)), the number of microspheres with holes on the surface increased and the holes tended to become larger. When reaching the 7th week (Figure 7(D1–D3)), due to extensive degradation and erosion of the microspheres as a whole, most of the microspheres broke up during the drying process and a large number of microsphere fragments could be observed (Chen et al., 2018). After 9 weeks of degradation, PLGA microspheres almost disappeared and only a few fragments were observed (Figure 7(E1–E3)).

### 3.4. *In vivo* pharmacokinetic study

**Figure 8.**
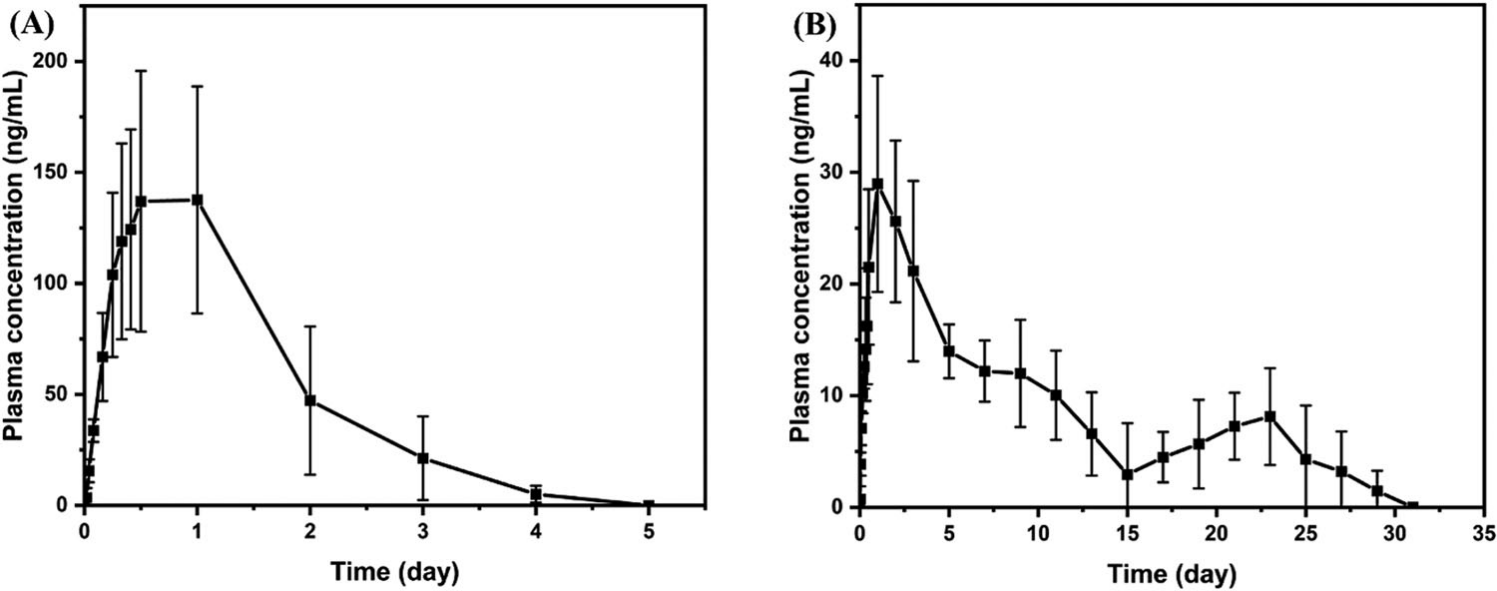
Mean plasma concentration–time profiles of BCL in rabbits. (A) Group I: oral administration of BCL (2.73 mg/kg) and (B) group II: intramuscular administration of BCL-loaded PLGA microsphere (BCL dose: 2.73 mg/kg) (mean ± SD, *n* = 6).

The mean plasma concentration–time distribution results of group I and group II in the rabbit model are shown in Figure 8. The pharmacokinetic parameters were analyzed using the non-compartmental model and listed in Table 2. The results showed that the MRT value of intramuscular injection of BCL-loaded PLGA microspheres was significantly increased (234.45 h) compared with those of the oral group, indicating that the BCL-loaded PLGA microsphere achieved the continuous release of BCL and maintained the concentration of BCL in plasma for up to one month *in vivo*.

**Table 2.** Main pharmacokinetic parameters of rabbits after oral BCL (2.73 mg/kg) and intramuscular injection BCL-loaded PLGA microspheres (BCL 2.73 mg/kg).

Parameter	Group I	Group II
*T*_max_ (h)	15.77 ± 6.77	36.00 ± 20.08
*C*_max_ (ng/mL)	151.18 ± 55.00	30.64 ± 9.10
AUC_last_ (h*ng/mL)	6054.29 ± 2626.12	6748.02 ± 2075.02
AUCINF-obs (h*ng/mL)	6276.17 ± 2626.66	7938.32 ± 1845.64
Vz/F-obs (mL/kg)	11570.36 ± 5251.75	81714.01 ± 73960.69
Cl/F-obs (mL/h/kg)	499.44 ± 164.52	396.84 ± 74.13
MRT_last_ (h)	28.70 ± 2.61	254.89 ± 48.58

As shown in Figure 8(B), the pharmacokinetic curves of BCL-loaded PLGA microspheres showed double peaks, which may be related to *in vitro* release characteristics. The plasma concentration of BCL reached the first peak at 36 h, which was mainly caused by the release of BCL attached to the surface of microspheres, and then began to decline slowly. This corresponded to the first stage of the *in vitro* release curve. At this stage, the release of BCL was mainly related to its dissolution characteristics, since the erosion of polymer was not obvious. The diffusion rate of BCL from the inside to the outside of the polymer was slow because it was classified as BCS II (low dissolution and high permeability). Therefore, the elimination rate of BCL *in vivo* was greater than the release rate at this stage. The concentration of BCL in plasma began to rise again on the 15th day after administration, and a second peak appeared on the 23rd day. This corresponded to the second stage of *in vitro* release. BCL accumulated in PLGA microspheres began to be released in large quantities due to the intensification of overall erosion of microspheres. The second peak in the pharmacokinetic curve occurred when the rate of release was equal to the rate of elimination *in vivo*. Moreover, it could also be found that the drug release duration *in vivo* was nearly 10 days shorter than *in vitro*, which might be the presence of biological components *in vivo* such as enzymes (Lu et al., 2014) to accelerate the degradation of PLGA.

## 4. Conclusions

In this study, a three-phase focused microfluidic device was developed to prepare PLGA microspheres to develop a sustained-release drug delivery system for insoluble small-molecule drugs. PLGA microspheres prepared by microfluidic technology had smooth surfaces, uniform particle size, and high monodispersity. We also studied the effects of different preparation parameters on DL% and EE%, including polymer concentration, drug concentration, and internal solvent ratio, to develop optimal prescription. *In vitro* release and degradation studies showed that the mechanism of drug release was driven by both drug diffusion and polymer erosion. *In vivo* pharmacokinetic results showed that the drug could be released continuously *in vivo* for up to 30 days, demonstrating the sustained release ability of PLGA microspheres. Therefore, the microfluidic device in this study is a simple and convenient device for the development of uniform monodisperse microspheres. PLGA microspheres prepared by this method and the prescription process can be used as a promising dosage form choice for insoluble small-molecule drugs to improve the potential utility of drugs.
